# Advances in the Involvement of Gut Microbiota in Pathophysiology of NAFLD

**DOI:** 10.3389/fmed.2020.00361

**Published:** 2020-07-29

**Authors:** Xiaofan Jiang, Juan Zheng, Shixiu Zhang, Baozhen Wang, Chaodong Wu, Xin Guo

**Affiliations:** ^1^Department of Nutrition and Food Hygiene, School of Public Health, Cheeloo College of Medicine, Shandong University, Jinan, China; ^2^Department of Endocrinology, Union Hospital, Tongji Medical College, Huazhong University of Science and Technology, Wuhan, China; ^3^Hubei Provincial Clinical Research Center for Diabetes and Metabolic Disorders, Wuhan, China; ^4^Department of Nutrition, Texas A&M University, College Station, TX, United States

**Keywords:** circadian, microbiota, metabolic diseases, NAFLD, inflammation, reprogramming

## Abstract

Non-alcoholic fatty liver disease (NAFLD) is characterized by hepatic steatosis and progresses to non-steatohepatitis (NASH) when the liver displays overt inflammatory damage. Increasing evidence has implicated critical roles for dysbiosis and microbiota-host interactions in NAFLD pathophysiology. In particular, microbiota alter intestine absorption of nutrients and intestine permeability, whose dysregulation enhances the delivery of nutrients, endotoxin, and microbiota metabolites to the liver and exacerbates hepatic fat deposition and inflammation. While how altered composition of gut microbiota attributes to NAFLD remains to be elucidated, microbiota metabolites are shown to be involved in the regulation of hepatocyte fat metabolism and liver inflammatory responses. In addition, intestinal microbes and circadian coordinately adjust metabolic regulation in different stages of life. During aging, altered composition of gut microbiota, along with circadian clock dysregulation, appears to contribute to increased incidence and/or severity of NAFLD.

## Introduction

Non-alcoholic fatty liver disease (NAFLD) has become a leading cause of chronic liver disease worldwide. There are 25% of population in the world suffering from NAFLD, including children, adolescents, and elderly (1). NAFLD is characterized by hepatic steatosis. When exhibiting inflammatory damage and fibrosis in addition to steatosis, NAFLD progresses to non-alcoholic steatohepatitis (NASH), the advanced form of NAFLD. As supported by the results from various epidemiological and clinical studies, NASH is a causal factor of terminal liver diseases including liver cirrhosis and hepatocellular carcinoma. Unhealthy nutrition-related metabolic disorders, such as central obesity, insulin resistance, dyslipidemia, and hypertension are closely associated with NAFLD (2). Although the etiology and progression of NAFLD remain to be elucidated, growing studies indicate that, additional to insulin resistance and inflammation, gut microbiota, and circadian rhythmicity of hepatic metabolic genes are considered to play key roles in the pathogenesis of NAFLD (3, 4).

The gut microbiota is composed of huge numbers of microbes. Half century ago, it was discovered that the toxicity of *Escherichia coli's* endotoxin fatality rate was determined by the administering time of endotoxin (5). This phenomenal finding and others led to validation that the microbiota colonized within the gastrointestinal tract undergoes circadian oscillations, which influence the composition and function of gut microbiota (6, 7). For instance, the diurnal interaction between oscillating hosts and their gut microbiome affect the circadian clock activities in other tissues and organs (8, 9), which in turn critically regulate host's metabolic homeostasis (10).

It has been accepted that the intestine and the liver are closely linked. This link is manifested by that gut microbiota and its metabolites play critical roles in the pathogenesis of NAFLD. Also, circadian rhythm was reported to maintain hepatic glucose and lipid metabolic homeostasis through regulating gut microbiota balance. In this review, we focused on the regulation of gut microbiota in relation to hepatic lipid metabolism and liver function, the alterations of gut microbiota in NAFLD, and the effects of microbiota metabolites on the development of NAFLD. Furthermore, we evaluated the relationships among circadian clock, gut microbiota, and metabolic disease (in particular NAFLD). We also summarized the effects of intestinal microbes on regulating metabolism through reprogramming circadian clock. Lastly, we summarized the effects of the interplays between intestinal microbes and circadian on metabolism and NAFLD aspects in different stages of life.

## Gut Microbiota and Liver Pathophysiology

**Figure 1:**
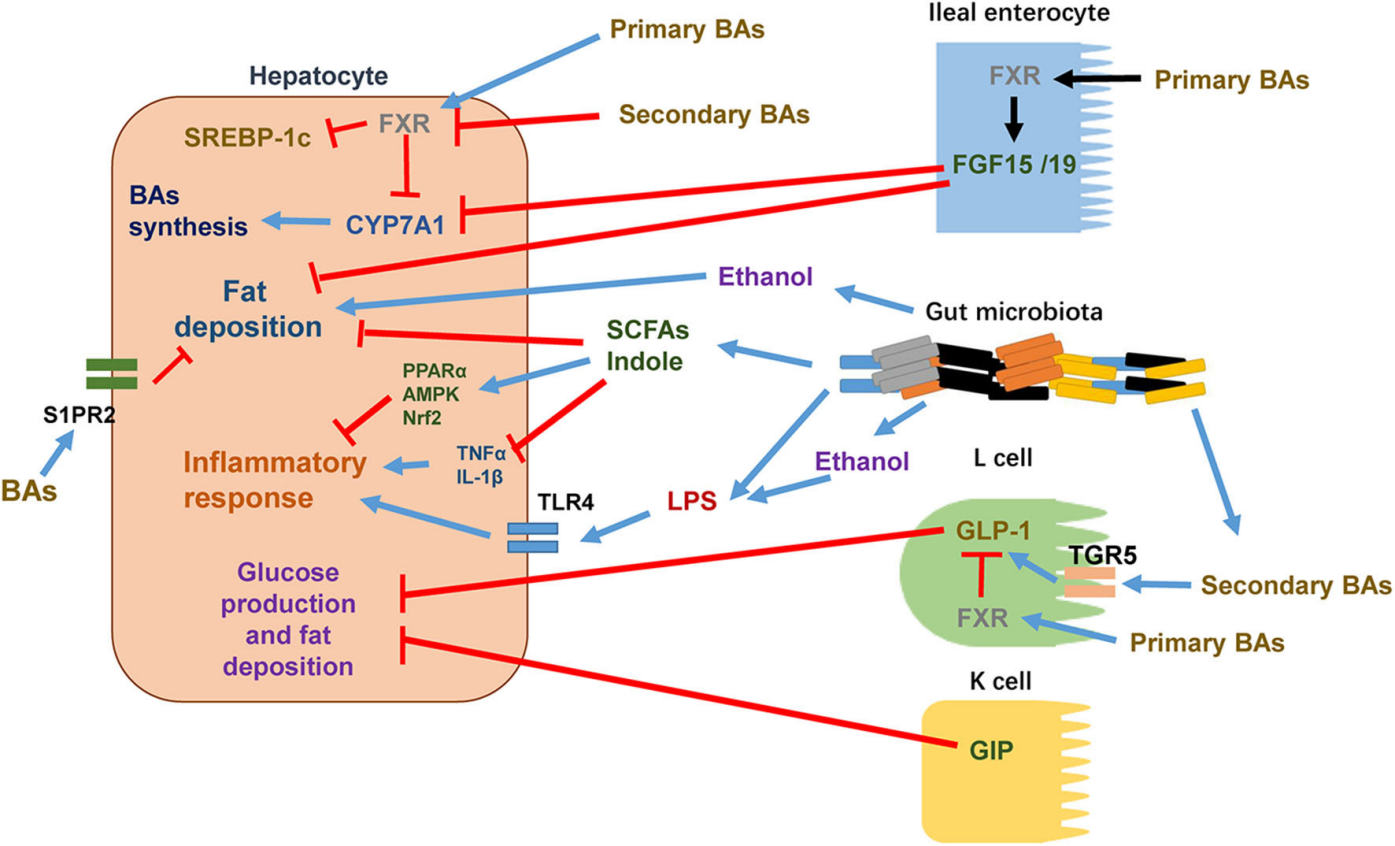
The crosstalk between intestine and liver in the pathophysiology of NAFLD. Certain intestine hormones, e.g., GLP-1 and GIP, reduce hepatic glucose production and fat accumulation. In L cells, secondary BAs stimulate GLP-1 synthesis and release via TGR5 activation whereas primary BAs activate FXR to inhibit GLP-1 synthesis and release. Other intestine hormones, e.g., FGF15 and FGF19, decrease hepatic lipogenesis. BAs stimulate FXR in ileal enterocytes, leading to the release of FGF15/19 into circulation. After reaching to hepatocytes, FGF15/19 suppresses BA synthesis through inhibiting CYP7A1 expression. Increased gut permeability, altered composition of gut microbiota, and elevated levels of gut microbiota metabolites such as ethanol are shown to enhance hepatocyte fat deposition and increase the flow of LPS into the circulation to promote proinflammatory responses through activating TLR4 signaling pathway in target cells. In hepatocytes, certain primary BAs acts through activating FXR to suppress the activity of SREBP-1c and thus reduces the expressions of lipogenic genes. Primary BAs also inhibit CYP7A1 expression and thus reduces BAs synthesis. Certain secondary BAs inhibit the activation of hepatic FXR. In addition to activation of FXR, BAs are shown to regulate hepatic lipid and sterol metabolism through activating S1PR2. Certain gut microbiota metabolites such as SCFAs and indole reduce hepatocyte fat deposition and proinflammatory responses via decreasing TNFα and IL-1β and/or activating PPARα, AMPK, and Nrf2.

Many studies have revealed that gut microbiota dysbiosis is linked to NAFLD (11, 12). The composition of gut microbiota varies from simple steatosis to NASH, fibrosis, and cirrhosis. Therefore, gut microbiota may be useful as predictors for NAFLD progression and severity (13, 14). Gut microbiota is capable of fermenting indigestible carbohydrates, resulting in important metabolites, such as short-chain fatty acids. The gut microbiota can also ferment tryptophan to generate other metabolites such as indole and indole derivatives. Animal studies and human studies have shown that these metabolites have beneficial effects on preventing against and/or alleviating obesity and NAFLD (15, 16). Understanding the mechanisms of how gut microbiota and metabolites are involved in NAFLD pathophysiology can inspire us to find out potential strategies to prevent or treat NAFLD/NASH. Recent advances in understanding the crosstalk between the gut and the liver pertinent to NAFLD pathophysiology is summarized in Figure 1 and detailed below.

### Influences of the Gut on Liver Metabolism

The intestine digests foods and absorbs nutrients. The liver receives nutrients from the intestine. As such, there are many metabolic events exhibiting the crosstalk between the gut and the liver. For instance, gut hormones participate in hepatic metabolism. In response to feeding, glucagon-like peptide 1 (GLP-1), which is secreted by the L cells of the small intestine, stimulates pancreatic β islet cells to produce insulin. Also, GLP-1 acts on GLP-1 receptor, present on human hepatocytes, to reduce hepatic glucose production and ameliorate hepatic fat deposition and insulin resistance (17). The release of intestinal GLP-1 enhances energy expenditure, which is associated with increases in the peripheral utilization of triglycerides (TG) for energy production, and reduces hepatic steatosis in mice fed a high-fat diet (HFD) (18, 19). Insulin-like peptide 5 (INSL5), which is also an L cell-derived gut hormone and regulated by gut microbiota, is reported to influence hepatic glucose production. Compared to that in conventionally raised (CONV-R) mice, the expression of INSL5 in the gut was 80-fold higher in germ-free (GF) mice and 20-fold higher in antibiotics-treated mice. The importance of INSL5 in regulating metabolism is further supported by the finding that INSL5^−/−^ mice exhibited decreased hepatic glucose production due to, in part, decreased expression of gluconeogenic enzymes such as glucose-6-phosphatase (G6Pase) and phosphoenolpyruvate carboxykinase (PEPCK) (20). In addition, glucose-dependent insulinotropic polypeptide (GIP), another gut hormone that is released from K cells located in the duodenum and proximal jejunum, regulates glucose homeostasis and lipid metabolism (21). Indeed, GIP appears to inhibit glucagon-stimulated hepatic glucose production through an indirect way (22). There is evidence suggesting that GIP influences hepatic insulin resistance and steatosis via regulating myeloid-cell-derived S100A8/A9 (23). Fibroblast growth factor 15 and 19 (FGF15 and FGF 19), which are also from the gut, were reported to ameliorate HFD-induced hepatic fat accumulation and ER stress (24). In particular, FGF 19 promoted hepatic glycogen and protein synthesis (25), reduced inflammation and fibrosis in liver injury mouse model through downregulating the expression of cholesterol 7α-hydroxylase (CYP7A1) and sterol-27-hydroxylase (CYP27A1) and thereby inhibiting bile acid synthesis (26).

Additional to gut hormones that regulate hepatic metabolism and inflammation, gut microbiota is associated with the development of NAFLD (27). For instance, gut permeability and small intestinal bacterial overgrowth are increased in patients with NAFLD compared with those in health controls. In this case, the increased gut permeability caused by alteration of intercellular tight junction likely contributes to the development and progression of NAFLD (28–30). Inflammation promotes the development of simple steatosis into NASH. In NAFLD, impaired intestinal barrier caused by nutrition stress increases the translocation of microbes and their products into the blood, leading to hepatic inflammation and even fibrosis/cirrhosis (31). Gut-derived antigens in the circulation are considered as major causing factors of strong inflammatory responses in the liver. Although intestinal permeability is not the main cause of liver inflammation and fibrosis, due to increased intestinal permeability, the inflammatory responses to microbial antigen strongly influence the progression of the disease.

Gut-derived bacterial products, such as lipopolysaccharides (LPS) and unmethylated CpG DNA, activate the signaling pathways involved in liver inflammation and fibrogenesis through stimulating innate immune receptors, e.g., Toll-like receptors (TLRs). In NASH patients, hepatic and serum TLR4 is significantly increased. Thus, high serum levels of TLR4 are considered as a bio-marker for liver fibrosis development (32). In a study involving TLR4-mutant mice, the results indicated that TLR4 was required for fructose to induce NAFLD. Compared with fructose-fed wild type mice, fructose-fed TLR4-mutant mice exhibited reduced hepatic fat accumulation, lipid peroxidation, inflammation, insulin resistance, and plasma ALT levels. This indicates the involvement of gut-derived endotoxin in the development of fructose-induced NAFLD (33). A similar study revealed that hepatic specific TLR4 deletion protected mice from fatty liver induced by 5% alcohol diet via decreasing the expression of hepatic inflammatory cytokines and endogenous lipogenesis (34). Saturated fatty acids (SFA) such as palmitate can activate proinflammatory signals through TLR4, inducing IL-1β and TNF-α production, as well as enhancing ROS production in hepatic infiltrating macrophages (35). Mechanistically, TLR4 promoting of the progression from simple steatosis to NASH involves in increases in ROS-dependent activation of X-box binding protein-1 (XBP-1) in Kupffer cells (36). TLR4 also is shown to induce transforming growth factor β (TGFβ) signaling pathway, activate hepatic stellate cell and increase extracellular matrix deposition, which all contribute to the progression of liver fibrosis (37). Moreover, gut microbiota and TLR4 appear to be required for the promotion of hepatocellular carcinoma (HCC), whose pathogenesis is enhanced by chronic liver inflammation and fibrosis (38).

More specific mechanisms of fat deposition and inflammation in the liver, caused by the alterations of gut permeability and barrier-induced infiltration of bacteria and bacteria products, involve increased signaling through nuclear factor kappa-light-chain-enhancer of activated B cells (NFκB) or c-Jun-N-terminal kinase (JNK), as well as increased levels of tumor necrosis factor alpha (TNFα) (39). Activation of NFκB in hepatocytes increased the production of cytokines and resulted in the recruitment and activation of Kupffer cells to mediate inflammation in the progression of NASH. Activation of NFκB induced the expression of TNFα, Fas ligand (FasL), and TGFβ, which contributed to fibrosis in NASH (40). Disruption of NFκB p65 in mice ameliorated HFD-induced hepatic steatosis and insulin resistance (41). JNK can be activated by diverse stimuli, such as cytokines, FFAs, reactive oxygen species (ROS), pathogens, and toxins. Activation of hepatic JNK decreased the expression of PPARα target genes and FGF21, up-regulated cytokines such as TNFα and interleukin-1 (IL-1), and promoted insulin resistance in liver (42).

### Regulation of Gut Microbiota by Hepatic Bile Acids

Primary bile acids (BAs), produced in the liver from cholesterol, serve as an emulsifier for lipid digestion in the intestine. Primary BAs become secondary BAs after being metabolized by intestinal flora. BAs are associated with the establishment of the gut microbiota; given that bile salts have anti-bacteria effects and only bacteria that are resistant to bile salts can survive in the intestine (43, 44). The antimicrobial actions of BAs are likely attributable to that BAs cause bacterial cell membrane damage through dissolving membrane lipids and dissociating membrane proteins. BAs also disturb macromolecular stability, such as misfolding or denaturing protein and inducing DNA damage and oxidative stress (44). Moreover, in human, chenodeoxycholic acid (CDCA) and cholic acid (CA), which are primary bile acids, as well as deoxycholic (DCA) and lithocholic acid (LCA), which are the predominant forms of secondary bile acids, activate the nuclear receptor farnesoid X receptor (FXR) to induce the expression of genes that are responsible for inhibition of microbial overgrowth and intestinal mucosal damage (45). It is known that BAs play an important role in regulating the composition of gut microbiota in response to diet. When mice consumed a Western diet, the profiles of BAs were altered, which increased Firmicutes, decreased Bacteroidetes, and disturbed the ecological balance of microbes (46). A similar study using FXR-deficient mice upon HFD feeding also revealed that the abundance of Firmicutes was increased and the abundance of Bacteroidetes was reduced. The profiles of BAs were featured by increased levels of primary bile acids such as beta-muricholic acids (βMCA) and taurine-conjugated beta-muricholic acids (TβMCA) and decreased levels of secondary bile acids such as ωMCA, hyodeoxycholic acid (HDCA), and hyocholic acid (HCA) (47). A rapid increase in the gut BAs pool (35 out of 42 quantified BAs) was observed in mice upon HFD feeding within 12 h, and an alteration in gut microbiota composition occurred at 24 h. Treatment of chow diet-fed mice with glycine-conjugated cholic acid (GCA) and taurine-conjugated cholic acid (TCA) increased obesity-related microbial population and brought about obese phenotype. Inhibition hepatic BAs synthesis in HFD-fed mice ameliorated HFD-induced dysregulation of microbial composition (48). In NASH-HCC mouse model, HFD accelerated the incidence of liver tumors, which was accompanied with increased the levels of hepatic BAs, including GCA, TCA, and taurochenodeoxycholate (TCDCA). The changes in gut microbiota were correlated with altered levels of BAs in the liver, suggesting that high hepatic BAs are associated with the dysregulation of gut microbiota and the development of HCC (49). Compared with those in healthy controls, fecal total and secondary BAs (LCA and DCA) were lower while primary BAs (CA and CDCA) were higher in patients with advanced cirrhosis. Patients with advanced cirrhosis also exhibited higher levels of Enterobacteriaceae and lower levels of Lachonospiraceae, Ruminococcaceae, and Blautia. Therefore, the amounts of primary and secondary BAs are associated with the population of key gut microbiota during the pathogenesis of cirrhosis (50). There also is evidence indicating that feeding mice high-saturated fats (from milk), compared to polyunsaturated fats or chow diet, resulted in alterations of BAs composition with increased levels of TCA and changes in gut microbiota with enhanced the abundance of Bilophila wadsworthia (51). IL-10^−/−^ mice on chow diet treated with TCA for a week exhibited higher abundance of Bilophila wadsworthia, which showed the similar results found in milk fat fed mice (52).

### Modulation of Bile Acid Metabolism by Gut Microbiota

Gut microbiota regulates the metabolism of BA synthesis. Compared with GF mice, the BA pool (mainly for conjugated and unconjugated βMCA) in CONV-R mice was reduced by 71%. The composition of BAs between CONV-R and GF mice was quite different in the cecum and colon. In the liver, CONV-R mice had higher levels of TCA and TαMCA and lower levels of TβMCA, compared with GF mice. The expression and activity of CYP7A1, which is a rate-limiting enzyme in BA synthesis in the liver, were downregulated in CONV-R mice. Furthermore, in FXR-deficient CONV-R mice, the levels of CYP7A1 were not decreased in the liver. Treatment of GF mice with FXR agonist INT-747 reduced the level of hepatic CYP7A1. These findings suggest that gut microbiota suppresses CYP7A1 expression in the liver in an FXR-dependent manner. Ileum FGF15 was involved in the regulation of CYP7A1 expression through FXR signaling. Treatment of CONV-R mice with antibiotics (bacitracin, neomycin, and streptomycin) suppressed FGF15 expression in ileum and enhanced the expression of CYP7A1, thus increasing the levels of primary BAs (TCA and TβMCA) and decreasing the levels of secondary BAs (DCA and ωMCA) (53). Besides regulating CYP7A1, gut microbiota also affects other key enzymes in the alternative pathway of BA synthesis such as oxysterol 7α-hydroxylase (CYP7B1) and CYP27A1 (45). In addition, gut microbiota not only regulates BA synthesis, but also modulates BA conjugation and reabsorption. Bile acid acyl-CoA-synthetase (BACS), which catalyzes taurine conjugation in BAs in the liver and apical bile acid transporters in the ileum, were downregulated in CONV-R mice (53). In a human study for chronic hepatitis B, the levels of total and primary BAs (TCDCA, GCDCA, GCA, and TCA) were upregulated in hepatitis B patients with moderate/advanced fibrosis, accompanied with downregulation of gut microbiota (such as Bacteroides and Ruminococcus) responsible for BAs metabolism (54). Trimethylamine N-oxide (TMAO), which is a metabolite produced by gut microbiota from choline, stimulated the expression of CYP7A1 in the liver, increased the serum levels of BAs and promoted FXR-antagonistic BAs (55).

### Altered Composition of Gut Microbiota During NAFLD

The composition of gut microbiota is altered during NAFLD. For instance, Lactobacillus species and some phylum Firmicutes such as Lachnospiraceae, genera, Dorea, Robinsoniella, and Roseburia were high in obese patients with NAFLD (56). Additionally, non-obese patients with NAFLD exhibited increased phylum Bacteroidetes and gram-negative bacteria and decreased Firmicutes including short-chain fatty acids-producing and 7α-dehydroxylating bacteria compared with healthy controls (57). When dietary choline was deficient, the levels of Gammaproteobacteria and Erysipelotrichi were correlated with the changes of fat accumulation in the liver. Gut microbiota such as Gammaproteobacteria and Erysipelotrichi can serve as a predictor for choline deficiency-induced fatty liver (58). Compared with that in NAFLD and healthy controls, higher abundance of Fusobacteria and Fusobacteriaceae was observed in NASH patients (59). Gut microbiota is related to advanced fibrosis in NAFLD. In both mild/moderate NAFLD and advanced fibrosis, the abundance of Firmicutes and Bacteroidetes is much higher. Proteobacteria is higher in advanced fibrosis, while Firmicutes is higher in mild/moderate NAFLD. Eubacterium rectale and Bacteroides vulgatu are rich in mild/moderate NAFLD, while *B. vulgatus* and *Escherichia coli* are rich in advanced fibrosis (60). Different steatosis in NAFLD patients exhibit differential compositions of gut microbiota. The abundance of Bacteroidetes is lower and the abundance of *C. coccoides* is higher during steatosis with inflammation and/or fibrosis, compared to simple steatosis (61). The composition of gut microbiota predicts the severity of NAFLD. Bacteroides is significantly higher in NASH and is independently associated with NASH, whereas Ruminococcus is higher in significant fibrosis (14).

### Microbiota Metabolites in the Pathophysiology of NAFLD

In addition to gut microbiota, microbiota metabolites also influence the pathophysiology of NAFLD. As it is established, microbial products derived from fermentation of dietary fiber and protein can affect liver metabolism and the development of NAFLD (62). Microbial metabolites are different during the progression from NAFLD to fibrosis. In advanced fibrosis, 3-phenylpropanoate, generated from anaerobic bacteria, is the mostly increased metabolite (63). Further analyses of proteins and enzymes indicate that the enzymes related to lactate, acetate, and formate are enhanced in mild/moderate NAFLD whereas the enzymes associated with butyrate, D-lactate, propionate, and succinate are increased in advanced fibrosis (60). The following microbiota metabolites are investigated mostly and closely related to NAFLD.

#### Short Chain Fatty Acids

Indigestible carbohydrates are fermented by gut microbiota and generate short chain fatty acids (SCFAs) such as acetate, butyrate, and propionate. Pectin, which is one of the soluble dietary fibers, is reported to prevent NAFLD in HFD-fed mice. Pectin increases acetic acid and propionic acid, as well as the levels of Bacteroides, Parabacteroides, Olsenella, and Bifidobacterium in the gut of HFD-fed mice (64). Gut-derived SCFAs such as propionate and acetate are metabolized by the liver and alter hepatic glucose and lipid metabolism (16). Serum metabolomics reveals that the serum levels of butyric acid and propionic acid were decreased in patient with NAFLD (65). Also, down-regulation of SCFA-producing bacteria contributes to increased energy intake and HFD-induced hepatic steatosis (66). Butyrate is reported to maintain intestinal mucosal health, including serving as a fuel source and regulating the immune system (67). There is evidence suggesting that butyrate ameliorates HFD-induced NAFLD and NASH via restoring the dysbiosis of gut microbiota and improving gut barrier (68), activating peroxisome proliferator-activated receptor alpha (PPARα) in the liver, suppressing hepatic inflammation and enhancing GLP-1R expression (69, 70). Moreover, butyrate-producing probiotic reduces hepatic lipid accumulation and inflammatory responses and improves hepatic insulin resistance via activating AMP-activated protein kinase (AMPK), AKT, and the expression of nuclear factor erythroid 2-related factor 2 (Nrf2) in rats with NAFLD (71). As supported by the results from a study involving G protein-coupled receptor 41 (GPR41)-deficient and GF mice, SCFAs binding to GPR41 may account for the regulation of gut microbiota, thereby host fat accumulation (72). Another study indicated that SCFAs acted through downregulating the expression levels of NLPR3, apoptosis-associated speck like proteins (ASC), and Caspase-1 to decrease inflammation in a manner involving G protein-coupled receptor 43 (GPR43) (73). Also, supplementation of SCFAs reduces hepatic fat deposition and inflammation by decreasing the activities of fatty acids synthases, increasing lipid oxidation via activation of AMPK, and suppressing the expression hepatic inflammatory cytokines such as interleukin-6 and TNFα (74, 75). SCFAs may also act through stimulating the release of GLP-1 to bring about beneficial effects on reducing fat accumulation and increasing insulin resistance (76).

#### Ethanol

Gut microbiota dysbiosis increases intestinal ethanol levels, which is associated with the progression of NAFLD. In patients with NASH, elevated ethanol-producing bacteria increased blood ethanol concentrations that are considered to be the reason of enhanced oxidative stress and inflammation in the liver (77), through increasing gut permeability, decreasing gut barrier, and increasing the levels of LPS in the intestine. Similar mechanisms also lead to increased transportation of endotoxin to the liver (78). In addition, ethanol has a direct harmful effect on the liver, leading to steatosis, steatohepatitis, and fibrosis (79). Ethanol stimulation of hepatic fat accumulation is likely attributable to increased production of acetate, a substrate for the synthesis of fatty acids. In ob/ob mice, a model of obesity and NAFLD, the levels of intestinal bacteria-derived ethanol are increased. In addition, treatment of ob/ob mice with antibiotics ameliorates ethanol-induced fat deposition and inflammation in the liver (78). There are different microbes responsible for ethanol production responding to different carbohydrates from diet. Most of ethanol is produced by *S. cerevisiae, L. fermentum*, and *W. confusa* after consumption of glucose, whereas the highest amount of ethanol is produced by *S. cerevisiae* and *W. confusa* after consumption of fructose. Therefore, inhibition of these microbes may be a viable strategy to reduce ethanol production and, thereby preventing NAFLD, NASH, or fibrosis (80).

#### Bile Acids

Primary BAs are synthesized by the liver whereas secondary BAs are metabolized by gut microbiota. As such, BAs are also considered microbiota metabolites. After its metabolism by gut microbiota, BAs return to the liver via the enterohepatic circulation through transporters on ileal enterocytes and hepatocytes. BAs regulate BA homeostasis, glucose and lipid metabolism through FXR signaling in hepatocytes, ileal enterocytes, and colonic L cells. Primary BAs such as CDCA, CA, T(G)CDCA, and T(G)CA are FXR agonists. In the liver, FXR activation by BAs inhibits expression of the CYP7A1. In ileum, FXR activation induces the expression of FGF15/19, which goes to the liver and also inhibits the expression of CYP7A1 and suppresses BA synthesis. In colonic L cells, FXR activation suppresses the synthesis and release of GLP-1 (81). Some BAs are reported to be FXR antagonists, such as UDCA (secondary BAs in human) and Tα/βMCA (primary BAs in mice). Secondary BAs such as LCA and DCA act as signal molecules to regulate energy homeostasis, insulin signaling, and inflammation via Takeda G-protein-coupled receptor 5 (TGR5) in colon, adipose tissue, muscle, and bone marrow (12). A study revealed that TGR5 activation improved glucose tolerance, increased energy expenditure, and decreased hepatic steatosis in HFD-induced obese mice (18). Altering BA profiles via diet, probiotics, medication, or surgery is reported to reverse obese-related metabolic phenotypes such as NAFLD/NASH through modifying BA composition. The latter involves appropriate regulation of hepatic metabolism through FXR and metabolisms in other tissues through TGR5 (82). For example, TGR5 activation by secondary BAs (such as LCA and DCA) in colonic L cells stimulated the synthesis and release of GLP-1, which inhibited glucose production and fat accumulation in hepatocytes. In patients with NAFLD, the serum levels of primary and secondary BAs were high, which were accompanied with decreased activation of FXR, fibroblast growth factor receptor 4 (FGFR4)-mediated signaling and serum levels of FGF19. In addition, secondary BAs were increased in the intestine of patients with NAFLD via enhancing the metabolism of taurine and glycine (83). In patients with NASH, *de novo* biosynthesis of bile acids in the liver was increased compared with that in healthy controls. Furthermore, increased *de novo* biosynthesis of bile acids may be closely associated with gut microbiota dysbiosis in NASH (84). CA was reported to prevent hepatic lipid accumulation and VLDL secretion via activation of FXR to suppress the activity of SREBP-1c and thus downregulate the expression of lipogenic genes (85). In addition to activation of FXR, conjugated-BAs were shown to regulate hepatic lipid and sterol metabolism through activating sphingosine-1 phosphate receptor 2 (S1PR2) to trigger ERK1/2 signaling pathway, which directly or indirectly modulates transcription of many genes such as CYP7A1, SREBP1c, and ApoB-100 (86). S1PR2 activation was also associated with reducing macrophage infiltration, which is the characteristic in NASH and fibrosis (87). Of note, BAs and the gut microbiota closely interact with each other. On the one side, BAs directly suppress bacteria growth in the gut through the anti-bacterial effects of BAs. On the other side, certain intestinal bacterial such as *L. monocytogenes* encode bacterial bile salt hydrolase (BSH), which in turn degrades BAs and helps bacteria to resist BAs (44). Interestingly, up-regulating BSH in conventionally raised mice reduces weight gain, plasma cholesterol, and liver triglycerides by regulating the transcription of genes related to lipid and cholesterol metabolism such as peroxisome proliferator-activated receptor gamma (PPARγ), ANGPTL4, and ABCG5/8 (88). Therefore, reducing BAs by modulating gut microbiota appears to be a viable strategy to improve NAFLD.

#### Indole and Indole Derivatives

As a bacterial degradation product of tryptophan, indole exerts powerful anti-inflammatory effects on immune cells and enterocytes (89). Subsequently, there are studies that have explored the effects of several indole derivatives as it relates to NAFLD. In a mouse model with HFD-induced NAFLD, Choi et al. examined the effects of indole-3-carbinol (I3C) on NAFLD phenotypes and attributed the anti-steatotic effect of I3C, at least in part, to decreased expression of lipogenic genes (15, 89–91). Similarly, two recent studies have shown that treatment with indole-3-acetate (I3A) alleviated NAFLD phenotypes in mice (92, 93). At the cellular level, I3A decreased hepatocyte production of palmitate, which was weakened by inhibition of aryl hydrocarbon receptor (AhR, a proposed receptor that mediates indole actions) (92). Moreover, I3A decreased hepatocyte mRNA levels of fatty acid synthase (FAS) and SREBP1c, a key transcription factor of lipogenic gene expression (94, 95), implying that I3A has a suppressive effect on hepatic lipogenesis. Consistent with the anti-NAFLD effects of indole derivatives, indole, *per se*, has also been validated to ameliorate diet-induced NAFLD phenotype in mice. Specifically, treatment of HFD-fed mice with indole, via intraperitoneal injection, for 9 weeks caused significant decreases in HFD-induced insulin resistance, hepatic steatosis, and liver inflammation (93). The mechanisms underlying the beneficial effects of indole are attributable to that indole reduced HFD-induced expression of hepatic lipogenic genes such as SREBP-1, steraroyl coenzyme decarboxylase 1 (SCD1), PPARγ, acetyl-CoA carboxylase1 (ACC1), and glycerol-3-phosphate acyltransferase, mitochondrial (GPAM), decreased the hepatic levels of reactive oxygen species (ROS) and lipid peroxidation product such as malonaldehyde, enhanced the activity of superoxide dismutase (SOD), and reduced hepatic macrophage infiltration, monocyte chemoattractant protein-1 (MCP1) and TNFα levels (93).

**Figure 2:**
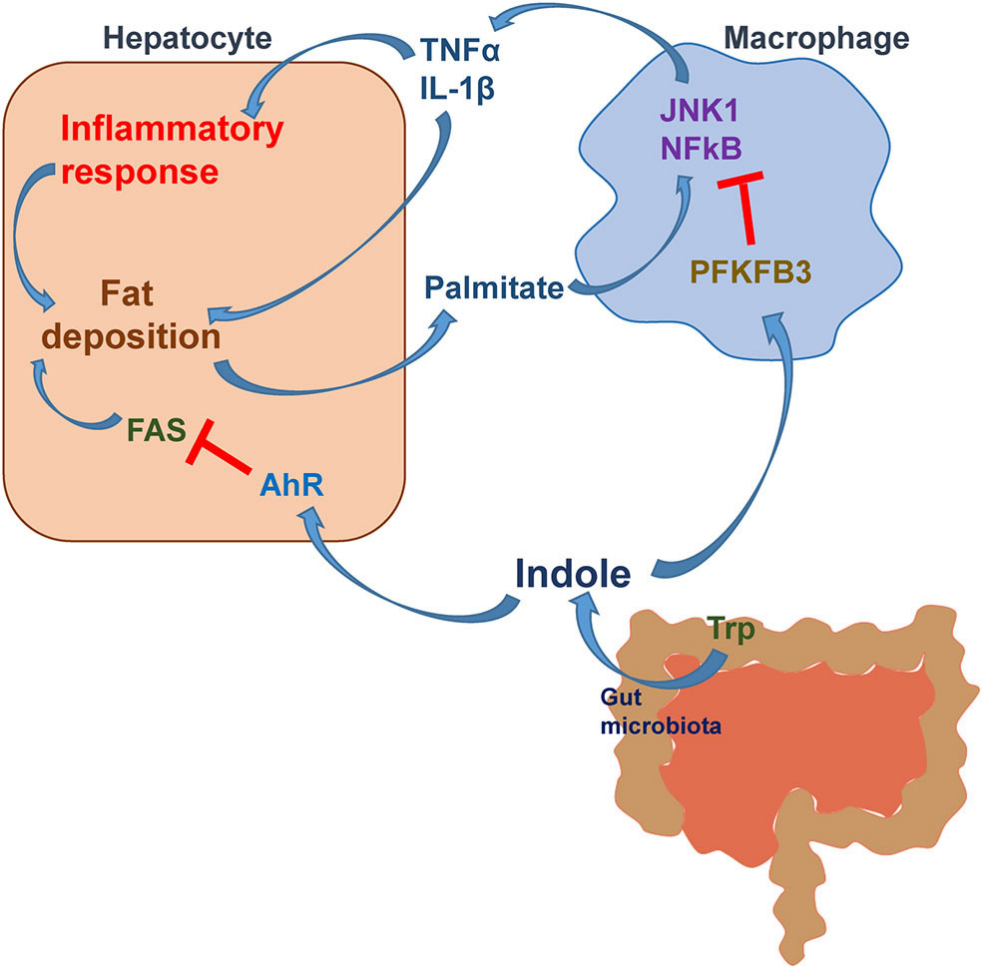
The mechanistic scheme for indole alleviation of NAFLD. During NAFLD, hepatocytes release fat deposition-associated proinflammatory mediators and palmitate (hydrolysis product of very low-density lipoproteins), which act on macrophages to enhance the proinflammatory responses. Active macrophages release proinflammatory factors such as TNFα and IL-1β and act, via paracrine manners, to exacerbate the proinflammatory responses and fat accumulation in hepatocytes. Indole, a microbiota metabolite from tryptophan (Trp), acts to reduce hepatocyte fat deposition via suppressing the expression of FAS through a mechanism involving AhR activation. Moreover, indole reduces the inflammatory responses in both macrophages and hepatocytes and fat deposition in hepatocytes in a manner involving myeloid cell PFKFB3. *Modified based on Krishnan, S., et al. Cell Reports, 2018. 23(4): p. 1099-1111 and Zheng et al. Front Med 2015; 9: 173-186*.

The relevance of indole to human NAFLD has been recently revealed, for the first time, in the study by Ma et al. In a cohort of 137 Chinese subjects, the circulating levels of indole were significantly lower than those in lean subjects and were reversely correlated with liver fat content (96). In parallel, the data from mice with diet-induced NAFLD further reveal that the hepatic levels of indole in HFD-fed mice were significantly lower than those in control mice. These two lines of evidence enabled the scientific premise for examining the effect of indole supplementation on alleviating NAFLD phenotype. As expected, oral supplementation of indole caused significant decreases in the severity of HFD-induced hepatic steatosis and inflammation. While gaining the mechanistic insights of indole actions, the study by Ma et al. also reveals that myeloid cell-specific disruption of PFKFB3, a master regulatory gene of glycolysis, nearly blunted the effects of indole on decreasing HFD-induced hepatic steatosis and inflammation. PFKFB3 is the gene encoding inducible 6-phosphofructo-2-kinase (iPFK2) (97), whose product fructose-2,6-bisphosphate is the most potent activator of glycolytic enzyme 6-phosphofructo-1-kinase (98–100). In macrophages differentiated from bone marrow cells, indole displayed a suppressive effect on LPS-induced proinflammatory responses in a PFKFB3-dependent manner (Figure 2). Moreover, hepatocytes co-cultured with PFKFB3-disrupted macrophages displayed increases in palmitate-induced fat deposition and LPS-induced proinflammatory responses. Of note, treatment with indole did not alleviate these responses in hepatocytes co-cultured with PFKFB3-disrupted macrophages as did it in hepatocytes co-cultured with control macrophages. Clearly, indole exerts an anti-NAFLD effect in a manner involving myeloid cell PFKFB3.

The study by Ma et al. also revealed a number of significant and interesting findings (96). In particular, mice with HFD-induced NAFLD revealed altered composition of gut microbiota relative to that in mice fed a control low-fat diet (LFD). Moreover, treatment of HFD-fed mice with indole brought about changes in the composition of gut microbiota in a manner similar to that in LFD-fed mice. This validates that indole, as a microbiota metabolite, also alters the composition of gut microbiota. Another important finding from the pharmacokinetic study is that indole reached its peak levels in the liver at 6 h post a single oral dosing of indole. In addition, the levels of indole were significantly higher than those in the circulation. Because of this, the liver is considered a primary organ where indole is metabolized. As such, the liver appears to be primary target for indole-based therapeutic approaches. The mechanistic scheme for indole actions is summarized in Figure 2.

Clearly, the intestine plays an important role in the pathophysiology of NAFLD. Intestine hormones, intestine conditions (such as permeability and intercellular tight junction), gut microbiota composition and balance, and microbiota metabolites regulate glucose production, lipogenesis, inflammatory response and insulin resistance in the liver by directly or indirectly ways. These advances have significantly improved our understanding of how the crosstalk between intestine and liver critically regulates the pathogenesis of NAFLD.

### Management of NAFLD/NASH via Modulating Gut Microbiota

As gut microbiota is considered to be a new therapeutic target for NAFLD/NASH, researchers are recently full of enthusiasm about looking for compounds to control NAFLD/NASH by altering gut microbiota. Probiotics are living microorganisms that can relieve intestinal diseases by restoring normal microbiota and provide health benefits to the host. A human study revealed that liver aminotransferases levels were improved in NAFLD patients treated with 500 million of *Lactobacillus bulgaricus* and *Streptococcus thermophiles* (101). MIYAIRI 588, a butyrate-producing probiotic from Japan, prevented hepatic steatosis from developing into liver cancer in a rat NAFLD model through activating of hepatic adenosine 5′-monophosphate-activated protein kinase (AMPK), AKT, nuclear factor erythoid 2-related factor 2 (Nrf2) and its targeted antioxidative enzymes (71). A probiotic mixture called VSL#3, which includes eight probiotic strains, has been proven to be very effective in the treatment of NAFLD. In obese children with NAFLD, supplementation with VSL#3 for 4 months decreased steatosis and BMI by enhancing the expression and the activity of GLP-1 (102, 103). Prebiotics, which are special form of dietary fibers, are fermented by gut microbiota to produce metabolites that promote the growth of beneficial intestinal flora. Alpha-galacto-oligosaccharides (alpha-GOS) from legumes was found to reduce food intake, improve fasting blood glucose, lower plasma non-esterified fatty acids, low-density lipoprotein (LDL), and total cholesterol in HFD-fed mice (104). Some phytochemicals also have prebiotic capacity and may become therapeutic compounds to prevent or treat NFALD. For instance, quercetin, which has antioxidant and anti-inflammatory properties, was reported to reduce hepatic fat accumulation, inflammation, and insulin resistance by increasing the population of Akkermansia genus in gut (105). Synbiotics, which are a combination of probiotics and prebiotics, was reported to provide more beneficial effects in NAFLD. Co-administering *Lactobacillus paracasei* N1115 and fructooligosaccharides in HFD-induced NAFLD mice reduced the levels of TNFα, insulin resistance and slowed the progression of cirrhosis (106). In lean patients with NAFLD, synbiotic (probiotics: 200 million bacteria of seven strains; prebiotic: 125 mg fructo-oligosaccharide) supplement significantly ameliorated fasting blood glucose, TG, and most inflammatory mediators (107).

SCFAs, which are metabolites from fermentation of dietary fiber by gut microbiota, have been used for preventing liver steatosis, inflammation, and fibrosis. Other metabolites such as BAs and indole-like molecules are potential therapeutic compounds to treat NAFLD/NASH. Antibiotics, such as neomycin and polymyxin B, can reduce fat accumulation in the liver by changing the gut microbiota and were found to be effective, to certain extent, for treating liver cirrhosis (108). After 90 days of solithromycin treatment, NASH patients showed reduction in liver steatosis and ALT levels (109). Gut-derived bacterial products and LPS increase hepatic inflammation in NAFLD through TLR4 signaling pathway. Blockage of TLR4 signaling pathway is considered as a potential therapy to alleviate hepatic inflammation and fibrosis. JKB-121, which is a TLR4 antagonist, was proved to reverse LPS-induced inflammation cytokine expressions, activation and proliferation of hepatic stellate cells, and collagen expression (110). Fecal microbiota transplantation (FMT) is an effective treatment for Clostridium difficile infection. There are some studies also suggesting that FMT may become a potential therapeutic strategy for NAFLD (111, 112). FMT from lean donors to obese recipients with metabolic syndrome for 6 weeks improved hepatic and systemic insulin sensitivity and increased butyrate-producing microbiota in obese recipients (113). A recent human study revealed that 6 weeks after allogenic FMT, small intestinal permeability in NAFLD patients was significantly reduced compared with that at baseline (114).

## Interplays of Circadian Clock and Gut Microbiota during NAFLD

There is evidence suggesting that circadian rhythms are related to gut microbiota, while gut microbiota also affects circadian rhythms (115). Both circadian and gut microbiota critically regulate metabolic homeostasis (116, 117) and are associated with the development of NAFLD (118, 119).

### Circadian Dysregulation and Gut Microbiota Dysbiosis

While highly relevant to human health, microorganisms in the human body maintain a dynamic balance in the body. Also, the circadian rhythm and the intestinal microbes are closely linked (120). Indeed, gut microbiota itself exhibits diurnal compositional and functional oscillations (121, 122). More specifically, environmental factors such as disruption of feeding time and sleep pattern are shown to impair microbiota diurnal rhythmicity and cause microbiota dysbiosis (122, 123). There also are studies showing that circadian disruption alters microbiota configuration in gut. For instance, disruption of BMAL1 in mice abolished the circadian rhythms of fecal microbiota in both sexes, while changing microbiota composition in a sex-dependent manner (6). Also, circadian CLOCK mutant mice exhibited lower evenness and diversity of gut microbiota compared with wild type mice when fed a chow diet. When mice were fed an alcohol diet, gut microbiota taxonomic levels in circadian CLOCK mutant mice were significantly different from those in wild type mice, indicating that gut microbiota community structure is altered (10, 124). Moreover, the circadian clock also alters the function of the gut microbes. As supporting evidence, the bacterial adhesion oscillation in PER1/2^−/−^ mice was remarkably disappeared (121). When combined with high-fat and high-sugar diets, mimicking rhythms disruption through frequent changes in light and darkness by reversing the light:dark cycle once weekly significantly changed the structure of microbial communities (124). Also, the microbe that impairs gut barrier integrity was increased and the microbe that improves the intestinal epithelial cell layer was decreased in mice exposed to constant 24 h light. Compared to that within normal light-dark cycles conditions, the diversity of rat's gut microbiota was significantly different in darkness or constant lighting conditions. The ratios of bacteria families such as Lactobacillus, Bacteroides, and Parabacteroides were altered in darkness or constant lighting conditions (125). In addition to the alterations of gut microbiota taxon, the expression of genes related to protective immune function was reduced whereas the expression of genes associated with gut inflammation was enhanced after circadian disruption. Specifically, the upregulated inflammatory genes include those for lipopolysaccharides (LPS) synthesis and transportation (126, 127).

Psychological factors also are shown to alter gut microbiota. A study reported that diurnal rhythm disorder caused by insomnia or a psychological and physiological pressure increased intestinal permeability and altered microbial composition (128). Also, in a study involving rhesus monkeys, stress was created by sound during pregnancy at night, and caused significant changes in intestinal microorganisms in the pregnant monkeys at 6 months before birth (129). The alteration of gut microbiota by stress includes reduced microorganism diversity and population of certain bacteria, such as Lactobacillus (130). Circadian rhythm disorder also can lead to the growth of some special intestinal microorganisms. In the first few days of sleep deprivation, mice revealed microbial invasion. At 20 days after sleep deprivation, the mice revealed 37 times more numbers of gram-negative bacteria in cecum relative to the control group (131). In a study involving human subjects, sleep deprivation increased Firmicutes in intestine, which usually found high relative abundance in obese population (132). The relative populations of Firmicutes, Lachnospiraceae, and Ruminococcaceae were increased and the relative populations of Bacteroidetes, Actinobacteria, Lactobacillmmaceae, and Bifidobacteriaceae were reduced in mice with 4 weeks of sleep fragmentation (133).

### Involvement of Gut Microbiota Dysbiosis in Circadian Disruption-Related NAFLD

“Time difference phenomenon” has destructive power and increases the tendency of illness (122). Microbial dysregulation caused by circadian rhythm disorder leads to an increased probability of metabolic diseases such as obesity, insulin resistance, and NAFLD (134–136). There are studies showing that germ-free mice did not respond to HFD feeding whereas normal mice with microbiota became obese when fed with HFD (7). HFD-feeding altered the oscillations of gut microbiota composition and function, which were associated with disturbed host circadian rhythm and led to host metabolic dysregulation (7). This finding is similar to that observed in the human after weight loss surgery. The latter revealed that the energy intake was decreased and the numbers of bacteria were changed (such as increased levels of Prevotella and Bacteroides and decreased levels of Firmicutes) after gastric bypass surgery (137). In addition, chronic sleep restriction is associated with metabolic diseases including NAFLD. Workers with constant shift in schedules or individuals with frequent jet-lag exhibit alterations in gut microbiota, leading to increasing inflammatory responses and metabolic diseases (138). Mice under the treatment of inverted dark-light every 2 weeks for 8 weeks, which mimicked shift work, exhibited significantly increased intestine permeability and altered community of gut microbiota, systemic insulin resistance, dyslipidemia, and inflammation (139). Transplanting microbiota from circadian disrupted (such as jet-lagged) human to germ-free mice increased weight gain and blood glucose levels (122), which are associated a significant increase in the incidence of NAFLD.

In the pathogenesis of NAFLD or the progression to steatohepatitis, intestinal microbiota composition exhibits altered circadian oscillation, which enhances the permeability of intestinal endothelial barrier, leading to intestinal and hepatic inflammation (122, 140). Moreover, gut microbiota is involved in the regulation of the expression of circadian clock genes in the liver. This is significant because hepatic circadian disorder is associated with hepatic lipid accumulation, inflammation, and oxidative stress (141). In a study involving mice with diet-induced obesity and NAFLD, time-restricted feeding (feeding only for 8 h during dark phase) for HFD-fed mice, which consumed the same amount calories as that of HFD *ad libitum* mice, altered hepatic clock genes that are related to key enzymes for glucose and lipid metabolism in the liver, thus decreasing hepatic fat accumulation (142). Mice fed an *ad libitum* HFD displayed alterations in gut microbiome, luminal metabolomics, gut signaling, and hepatic gene expression, which resulted in metabolic dysregulation such as obesity, impaired glucose metabolism, insulin resistance, hepatic steatosis, and inflammation. However, mice with time-restricted HFD feeding revealed decreased obesogenic microbiota, increased obesity-protective microbiota, enhanced carbohydrate excretion, restored gut signaling and hepatic gene expression, which appeared to protect against obesity and metabolic dysregulation (136). Circadian disruption (mimicking shift work or jet-lag) in rats enhances the inflammatory responses when treated with LPS. In particular, Kupffer cells (KCs) isolated from circadian disrupted rats exhibited increased TNFα expression in response to LPS, indicating that liver immune cells are modulated by circadian rhythms (143). Furthermore, KCs itself showed circadian oscillation, indicated by the findings that the numbers of KCs varied during the circadian cycle and that some proteins in KCs have diurnal rhythmicity. The connection between immune response proteins of KCs and liver immune proteins is dominant during the daytime whereas the connection of metabolic proteins between KCs and liver is dominant during the nighttime (144). A study in which HFD-fed mice were under constant light revealed that melatonin ameliorated HFD- and circadian disruption-induced hepatic fat accumulation and insulin resistance and restored the gut microbiota. The latter was evidenced by that melatonin reversed the increased ratio of Firmicutes to Bacteroidetes (145).

## Gut Microbiota Regulation of NAFLD During Aging

### Gut Microbes in Infants and Young Children

In an infant, the majority of bacterial strains comes from the mother. While most of the bacteria cannot be colonized for a long time (146), some intestinal strains always live with the host (146). It has been previously thought that baby's intestines are sterile. Numerous studies have now indicated that Staphylococcal and Enterococci are present in infant feces, verifying that microbial colonization has already occurred in the intestines (147). Compared with normal control, early intestinal microbiota in cesarean section infants is reduced and associated with T helper-1 (TH1) response (148). This in turn affects the weight of childhood; although the underlying mechanisms remain to be elucidated (149). In addition, premature infants with low birth weight exhibit altered intestinal microbes and increased risk of metabolic abnormalities (150). Accordingly, early control of multiple metabolic diseases, e.g., obesity (151) and diabetes (152), which both increase the incidence of NAFLD, may be achievable through breastfeeding. In severely malnourished children, microbes are lagging behind and cannot maintain optimal homeostasis, indicating that intestinal microbes play a role in metabolism (153). Gut microbes interplay with a variety of factors, including genetics and the environment (154). Congenital genetic materials can generate a significant impact on adults (155). Compared with those in normal mice, the numbers of gut microorganisms in the mice with congenital obesity have changed significantly (mainly Bactericides and Formicates) (156), and the alterations are also observed in human (157). Compared to healthy children, children with NAFLD have higher levels of Gammaproteobacteria and Prevotella, as well as higher levels of ethanol (158). In a similar study, the results indicate that children with NAFLD have higher levels of Actinobacteria and lower levels of Bacteroidetes compared with healthy controls. In addition, the levels of Bradyrhizobium, Anaerococcus, Peptoniphilus, Propionibacterium acnes, Dorea, and Ruminococcus are increased and the levels of Oscillospira and Rikenellaceae are reduced in children with NAFLD (159).

Both gut microbiota and circadian rhythms are linked to the metabolic homeostasis in infants and children and influence their health in the future. For instance, early microbial destruction induces metabolic dysregulation. Cho et al. found that treatment with antibiotics in early life in mice increased the levels of GIP, adiposity, and the expression of hepatic genes, which are involved in lipid metabolic processes. Although early antibiotics did not change the overall numbers of microbes, the composition of gut microbiota was altered in mice with antibiotics in early life, such as increased levels of Firmicutes (160). Of note, under a chow diet, limited antibiotics ameliorated hepatic accumulation of fat in early age in male mice (161). Mechanistically, LPS from gut microbiota is associated with the development of metabolic syndrome in children. A study indicates that sleep disruption contributes to gut bacteria dysbiosis and the increase in LPS levels, leading to inflammation and metabolic dysregulation (162). Child snoring disturbs sleeping pattern and is related to metabolic syndrome, neurocognitive, and behavioral problems. In the gut of children with snoring, the diversity of microbiota was reduced and pro-inflammatory bacteria population and the ratio of Firmicutes to Bacteroidetes were increased (163). Also, the results from a human study involving 40 children with NAFLD indicate that the serum levels of FGF21 were inversely associated with the severity of NAFLD in children at 8:00 am whereas more severe NAFLD revealed increased FGF21 levels at noon (164).

### Gut Microbes Regulation of NAFLD During Aging

From colonization in early life, the body maintains the balance of microbes for decades and toward the end of life. For elders, their intestinal tract is fragile, their teeth are loose, and there are other factors affecting the intestinal microbes (165). In an epidemiological survey (166), the results obtained suggest that total proteobacteria are increased and stable within a limited time in people over 65 years old. However, there are some differences between the studies about whether the diversity of Bactericides is increased (167). Some studies suggest that the diversity of Bactericides is increased (167) whereas others showed the opposite results (168). Also, the gut microbial composition appears to be different in a sex-dependent way in elders. Obese male elders have lower levels of Bacteroidetes than obese female elders (169). Moreover, Clostridium levels are different between elders and young adults. The production of short chain fatty acids is reduced in elders, compared with young adults (170).

The circadian rhythm controls deep sleep and duration (171). In the conventional consciousness, the elders have less deep sleep (172) and more awakening (173). Through detecting body temperature and melatonin rhythm (174), the phase of rhythm is shifted forward (175), the amplitude of rhythm is reduced (176), and PER2 expression is impaired in elders (177, 178). These findings have been confirmed by many clinical studies. Indeed, chronic sleep disorders in old adults are associated with metabolic dysregulation. Also, in elders, diet has more effects on age-related dysbiosis in gut microbiota that affects circadian rhythm in the host and exacerbates metabolic disorders (7). The circadian rhythm gradually deteriorates in life (179), characterized by the shortening of sleep time, the loss of circadian amplitude (180), the reduction of neuronal synapses (181), and the increase in the proportion of silent cells (182). However, the results of a human study suggest that the risk of NAFLD is increased slightly in a middle-aged and elderly Chinese population with a long night time sleep duration (183). Similarly, young and old mice fed an HFD for 12 weeks revealed increased body weight, fat accumulation, insulin resistance, and NAFLD activity score regardless of sex. However, old mice exhibited exacerbation of NAFLD severity and gut microbiota dysbiosis (184). In elder people, the numbers of protective anaerobic bacteria are reduced, gastrointestinal function is declining, and the severity of hepatic steatosis and inflammation is greater in response to HFD. As such, the health status of the elders should be taken serious consideration (185).

## Conclusion

**Figure 3:**
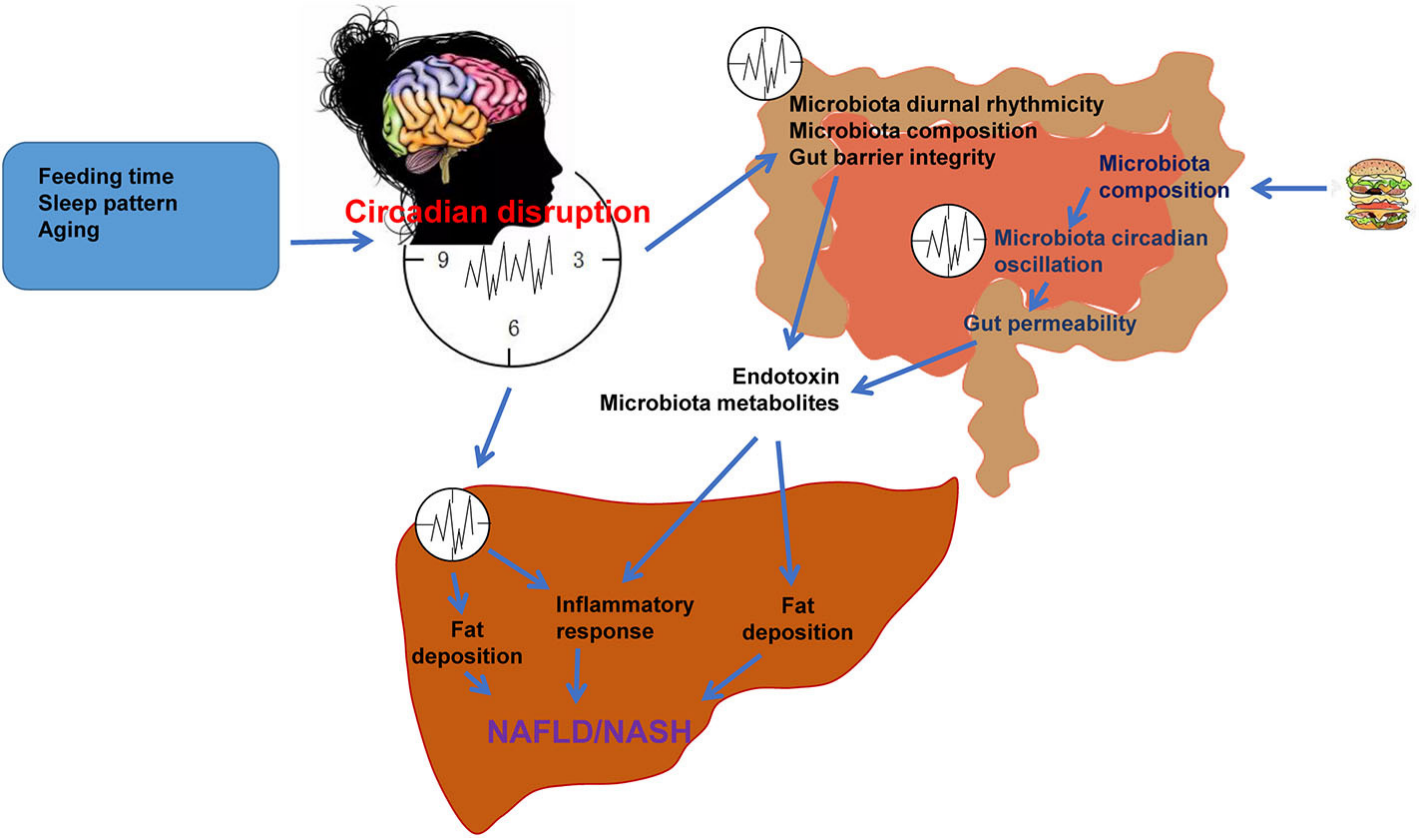
Circadian rhythms and gut microbiota in the pathogenesis of NAFLD. Under normal physiological conditions, the central and peripheral clocks operate synchronously to maintain the normal operation of the body. Feeding time, sleep pattern, and aging cause circadian dysregulation, leading to alterations of microbiota diurnal rhythmicity, microbiota composition, and thus microbiota dysbiosis. Microbiota dysbiosis impairs gut barrier integrity and increases the gut permeability, which results in increased release of microbiota products such as endotoxin and microbiota metabolites into circulation. HFD or nutritional stress changes microbiota composition and circadian oscillation, increasing gut permeability and release of microbiota products. These microbiota products reach the liver and cause hepatic steatosis and inflammation, which are the features of NAFLD/NASH. Circadian disruption also directly causes dysregulation of liver metabolism, promoting NAFLD/NASH through increasing hepatic fat accumulation and inflammation.

Nutrition, lifestyle and environment (day and night cycle) influence metabolism, thereby the health, life quality, and life span. Individuals who are shift workers, frequently cross-continental traveler undergoing jet-lag, suffers of sleep disorders, and/or frequent consumers of high-fat and/or high-sugar diets have increased risks for metabolic diseases including NAFLD and NASH. Pathologically, dysregulation of circadian rhythms, along with dysfunctional composition of gut microbiota contribute to the development and progression of NAFLD, which has been summarized by this review (Figure 3). There exist circadian rhythms in intestinal microbes. The changes in intestinal microbes' oscillation are manifested by increased intestinal permeability, microbial composition, and increased inflammation. Intestinal microbe regulates metabolism via reprogramming circadian clock, in particular the liver circadian clocks. Aging and unhealthy diet, as well as dysfunctional intestinal microbes are factors that bring about rhythm disorders, leading to hepatic fat accumulation and inflammation. As such, a healthy diet and a clocklike lifestyle are of the effective ways to prevent NAFLD and maintain metabolic homeostasis, thereby keeping individuals healthy.

## Author Contributions

XJ and XG wrote most of this review. JZ, SZ, BW, and CW wrote some sections. CW and XG made the final editing. XG came up with the concept. All authors contributed to the article and approved the submitted version.

## Conflict of Interest

The authors declare that the research was conducted in the absence of any commercial or financial relationships that could be construed as a potential conflict of interest.
